# Synaptic Symmetry Increases Coherence in a Pair of Excitable Electronic Neurons

**DOI:** 10.1371/journal.pone.0082051

**Published:** 2013-12-02

**Authors:** Bruno N. S. Medeiros, Mauro Copelli

**Affiliations:** Departamento de Fsica, Universidade Federal de Pernambuco, Recife, Pernambuco, Brazil; Universiteit Gent, Belgium

## Abstract

We study how the synaptic connections in a pair of excitable electronic neurons affect the coherence of their spike trains when the neurons are submitted to noise from independent sources. The coupling is provided by electronic circuits which mimic the dynamics of chemical AMPA synapses. In particular, we show that increasing the strength of an unidirectional synapse leads to a decrease of coherence in the post-synaptic neuron. More interestingly, we show that the decrease of coherence can be reverted if we add a synapse of sufficient strength in the reverse direction. Synaptic symmetry plays an important role in this process and, under the right choice of parameters, increases the network coherence beyond the value achieved at the resonance due to noise alone in uncoupled neurons. We also show that synapses with a longer time scale sharpen the dependency of the coherence on the synaptic symmetry. The results were reproduced by numerical simulations of a pair of synaptically coupled FitzHugh-Nagumo models.

## Introduction

Neurons are highly nonlinear dynamical systems which are typically connected to tens of thousands of other neurons, the whole system being subjected to fluctuations whose stochasticity cannot be dismissed. This interplay between nonlinearity, high dimensionality and noise is what renders the brain a difficult and interesting system to study [1], [2]. More generally, the last decades witnessed a surge in theoretical studies of collective phenomena of interacting nonlinear units. Since the seminal work of Kuramoto [3], for example, several aspects of synchronization have been addressed [4]. With the emergence of complex networks becoming a research topic in itself [5], the effects of topology on synchronization have been thoroughly investigated (see e.g. [6]–[8] for recent examples, or [9] for a review). Recently, even the notion of networks of networks have emerged in the context of climate studies [10].

Even single neurons, however, can reveal surprises. In 1997, for instance, Pikovsky and Kurths unveiled the phenomenon of coherence resonance (CR), whereby an excitable system driven by white noise produces a spike train whose regularity (or coherence) attains a maximum at some finite value of the noise intensity [11]. In the low-noise regime, the spike train approaches a Poissonian incoherent behavior with small firing rate, whereas in the high-noise regime incoherence coexists with a large firing rate. At the resonance, the spike train looks almost periodic, despite the fact that the system is in an excitable regime, not tonic.

The collective effects of coupling on CR were subsequently investigated. It was shown that global coupling, either by square pulses [12] or via electrical synapses (gap junctions) [13], can lead to network synchronization with strong coherence. It was also shown that a network of excitable elements can exhibit system size CR, where increasing the number of elements in system leads, at first, to an increase in global coherence, while very large networks have reduced global coherence [13], [14]. In addition, chemical synapses were shown to be better at increasing global coherence than gap junctions, even when the analyzed network contained only two neurons [14]. This highlights the importance of the characteristic times introduced by the chemical coupling in the post-synaptic response.

Our aim in this contribution is twofold. First and foremost, we depart from previous studies on CR in networks of model neurons in that all of them have focussed on the effects of some coupling among the units which was *uniform* across the network: Wang et al. have employed the same uniform coupling intensity among all pairs of Hodgkin-Huxley neurons [12]; Toral and Mirasso had a single variable to parameterize the gap junction conductance among their FitzHugh-Nagumo elements [13]; and Balenzuela and Garca-Ojalvo simulated Morris-Lecar systems with chemical and electrical synapses: in either case, the same coupling intensity that connected neuron *i* to neuron *j* was also applied from *j* to *i*. In other words, previous analyses of collective effects have dramatically reduced the dimensionality of coupling space by focussing on the single-axis projection of uniform coupling. This is a convenient choice to render parameter space scanning feasible, but not very realistic. In nature, mutually connected neurons most probably are not symmetrically coupled. Here we set forth to investigate what happens to the coherence of spike trains when the coupling among the units is not necessarily uniform. Specifically, we fully explore the space of coupling intensities by focussing on the analysis of the simplest network, namely, a pair of neurons. We will show that: 1) synaptic asymmetry can deteriorate the coherence of a pair of neurons that were previously uncoupled; 2) synaptic symmetry leads the system to a more coherent state, compared with the uncoupled case; 3) the effects of synaptic symmetry on the system coherence is strongly dependent on the characterstic time scale of the synapses.

Second, we go beyond numerical simulations by employing type-II-excitable [15] electronic neurons which are connected via electronic circuits that mimic chemical synapses (see Materials and Methods). These electronic neurons are set at the edge of their Hopf bifurcation, as previously described [16]. The use of such electronic circuits gives our results an additional degree of robustness due to the variability of the electronic components and influence of external effects (such as thermal noise), all of which contribute to a more biologic-like scenario of noise and heterogeneities. Finally, we briefly discuss the potential applications of our results beyond neuroscience, in other experimental setups where CR has been observed.

## Materials and Methods

### Electronic neurons and electronic synapses

**Figure 1:**
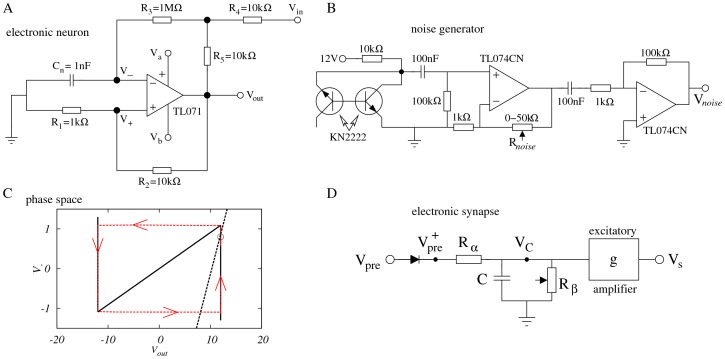
Electronic circuits. (A) Excitable electronic neuron circuit. 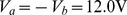 are the supply voltages, 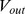 and 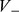 are the dynamic variables and 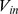 is an input voltage. 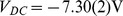. (B) Noise generator circuit. The noise intensity is controlled by the resistance 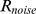. (C) Phase space diagram for the system described by Eqs. 1. The nullclines 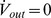 (black solid line) and 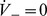 (black dashed line) intersect at the stable fixed point. Due to the noise provided by the noise generator at the input 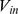, the system is forced out of the rest state, often having to perform a long excursion in the phase space, producing a spike (red line). (D) Circuit that mimics the behavior of a chemical synapse. 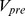 is the pre-synaptic input, while 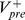 takes into account only positive values of 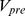. The resistances 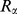, 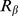 and the capacitor 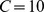 nF control the time scale 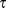 of the synapse (see text for details). The equivalent of the synaptic conductance 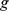 can be set by a standard amplifier, which yields the post-synaptic potential 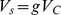 (see text for details).

Previously, we have introduced a FitzHugh-Nagumo-like electronic circuit which models neurons with type-II excitability, operating in the ms time scale and receiving a noisy input with controllable intensity [16]. The circuit diagrams for both the electronic neuron and the noise generator are shown in Fig. 1A and Fig. 1B respectively. The equations of motion that model the behavior of the electronic neuron can be readily obtained from Kirchoff's law and a simple model for the dynamics of the operational amplifier [16]: 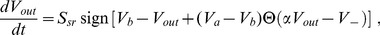
(1a)

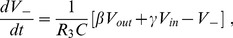
(1b)where 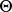 is the Heaviside function, *V_a_* and *V_b_* are the operational amplifier supply voltages and *α*  =  *R*_1_/(*R*_1_ + *R*_2_), *β*  =  *R*_4_/(*R*_4_ + *R*_5_) and *γ*  =  *R*_5_/(*R*_4_ + *R*_5_) (see Fig. 1A). The time scale for the dynamical variable *V_out_* is controlled by the slew rate *S_sr_* of the operational amplifier (Fig. 1A), with a typical value 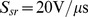. The characteristic time scale 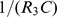 controls the dynamical variable *V_−_* and is set to yield *V_out_* as the fast variable and *V_−_* as the slow one. The input variable *V_in_* receives the sum of a constant DC signal *V_DC_*, a noisy signal *V_noise_* provided by the noise generator (independently for each electronic neuron) and the synaptic input *gV_C_* (see details below) which couples the two electronic neurons: 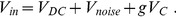
(2)


**Figure 2:**
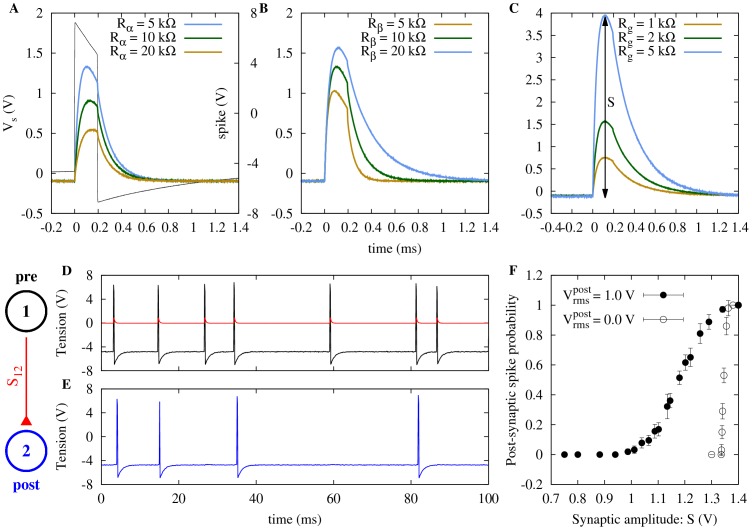
Output *V_s_* of the electronic synapse when subjected to a pre-synaptic spike. (A) Black line corresponds to the pre-synaptic spike (scale on the right). The rise time of 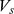 is governed by 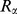 (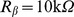, 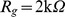), while (B) changes in both 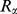 and 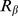 influence the decay time of 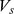 (when 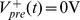; 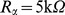, 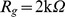). (C) The gain *g* (see text for details and Fig. 1) is proportional to the amplifier resistance 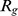, which affects only the maximum amplitude *S* of 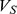 (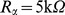, 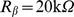). (D) Spike train of a pre-synaptic neuron excited by noise (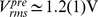) and the resulting 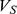 with 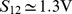 (red). (E) Spike train of a post-synaptic neuron (blue) excited by 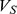 and a lower noise intensity (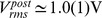). (F) Post-synaptic spike probability as a function of 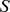 for two values of 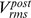. In both cases, the pre-synaptic neuron is subjected to 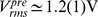.

The phase space of an uncoupled neuron (i.e. for Eqs. 1a and 1b with g = 0 in Eq. 2) is shown in Fig. 1C. The nullclines 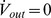 and 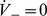 resemble those of the FitzHugh-Nagumo model (see below) and the single fixed point can have its stability changed via a Hopf bifurcation (controlled by the *V_DC_*) which generates a limit cycle and puts the system in a tonic regime. The membrane potential undergoing a spike (like the one showed in Fig. 2A, for instance), is obtained through a weighted subtraction of both dynamic variables, 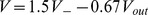. In the actual electronic neuron circuit this is achieved with the use of an analog subtractor [16], [17].

When set near its Hopf bifurcation, the electronic neuron is excitable, and its noise-induced spike train can be described approximately by the first two moments of the inter-spike interval (ISI) distribution 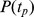 [11]. The incoherence of the spike train is characterized by the parameter 
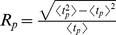
 and attains a minimum as a function of the noise intensity, which is controlled by the resistance 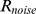 (Fig. 1B). Further details about the electronic neuron circuit, its model and the noise generator can be found in [16].

In order to connect two such electronic neurons, we have employed the electronic synapse shown in Fig. 1D. Due to the diode at its input, it is activated whenever the output 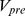 of the pre-synaptic neuron becomes positive (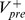), which happens only during a spike [16]. Applying Kirchoff's law to the circuit, one obtains the dynamics for the voltage 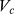 at the capacitor: 
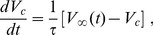
(3)where the characteristic time is 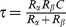 and the asymptotic value is 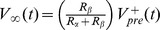. The electronic analog 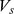 of the excitatory post-synaptic potential (EPSP) is obtained by multiplying 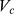 by a controllable gain with a standard amplifier [17] which effectively controls the strength of the coupling between the two electronic neurons: 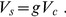
(4)


Here we focus on excitatory synapses (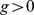), but an inhibitory synapse (i.e. with 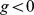) can easily be mimicked with an inverter amplifier. First-order kinetics such as that of Eq. 3 is considered a reasonable approximation for the dynamics of some classes of synapses (e.g. based on AMPA or GABA receptors) [2].

As shown in Fig. 2, this setup allows us to control several interesting features of the electronic EPSP: 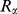 controls the rise time of 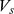 (Fig. 2A). Both 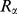 and 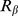 have influence on the decay time of 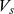, as well as on its maximal value 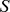 (Fig. 2B), which can also be independently controlled by the gain 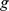 (Fig. 2C).

With a neuron in its excitable regime, we can control its spontaneous firing rate by adjusting the root mean square (rms) 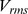 of the zero-mean noise voltage 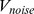 that stimulates it (i.e. 
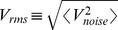
). Figure 2D shows a time series of a pre-synaptic neuron (labeled 1) with noise intensity 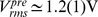 and the corresponding EPSPs it generates whenever it spikes. A post-synaptic neuron (labeled 2), also in its excitable regime, receives these EPSPs as well as noise, but with lower intensity 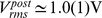 (noise sources in different neurons are independent). Driven by this lower-intensity noise alone, the post-synaptic neuron is extremely unlikely to fire, which it eventually does upon receiving an EPSP with peak voltage 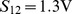 (see Figs. 2D and E). In Fig. 2F we show that the post-synaptic spike probability increases monotonically with the synaptic amplitude 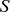, with a sensitivity threshold that decreases with increasing noise intensity 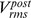.

In what follows, experimental results were obtained with electronic neurons whose parameters were chosen to be as similar as possible (within the ∼5% tolerance of the electronic components). Where not shown, error bars are smaller than symbol sizes and uncertainties in experimental values are in the range of 1%. The noise intensity will be denoted by the resistance 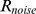 controlling the gain of the noise amplifier, as the voltage rms increases linearly with 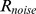 [16]. Experimentally, the peak value (amplitude) *S* of the EPSP 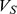 (see Fig. 2B) is easier to measure than the gain parameter 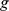, and will therefore be used as a measure of the synaptic strength, with 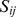 denoting the coupling from neuron *i* to neuron *j* (with 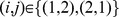, in the present case).

### Computer simulations

Numerical simulations using the FitzHugh-Nagumo model for neuronal excitability were made to confront the experimental results. This model was chosen due to its simplicity and close similarity with our model for the electronic neuron dynamics (see Eqs. 1 and 2): 
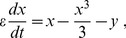
(5a)

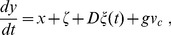
(5b)where 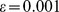 is set to reproduce the ratio of the time scales governing the variables in Eqs. 1 and 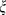 is an assumed delta-correlated Gaussian noise with zero mean whose intensity is controlled by the parameter *D*. Similarly to Eqs. 1 and 2, the strength of the synaptic coupling between the two model neurons is controlled by the gain constant *g*. The parameter 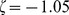 ensures that each FitzHugh-Nagumo model is in a excitable regime but very close to its Hopf bifurcation [11]. Coupling between the two FitzHugh-Nagumo systems is achieved using the model for the electronic synapse, as previously described in Eq. 3: 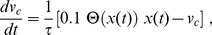
(6)where again 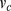 integrates pre-synaptic activity *x* only when it is positive. We will employ the gain 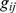 as a measure of the coupling strength from model neuron *i* to model neuron *j*. The equations were integrated using Euler-Maruyama's method with a time step 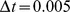.

## Results and Discussion

### Coherence deteriorates with an incoming synapse, but is restored with an outgoing synapse

**Figure 3:**
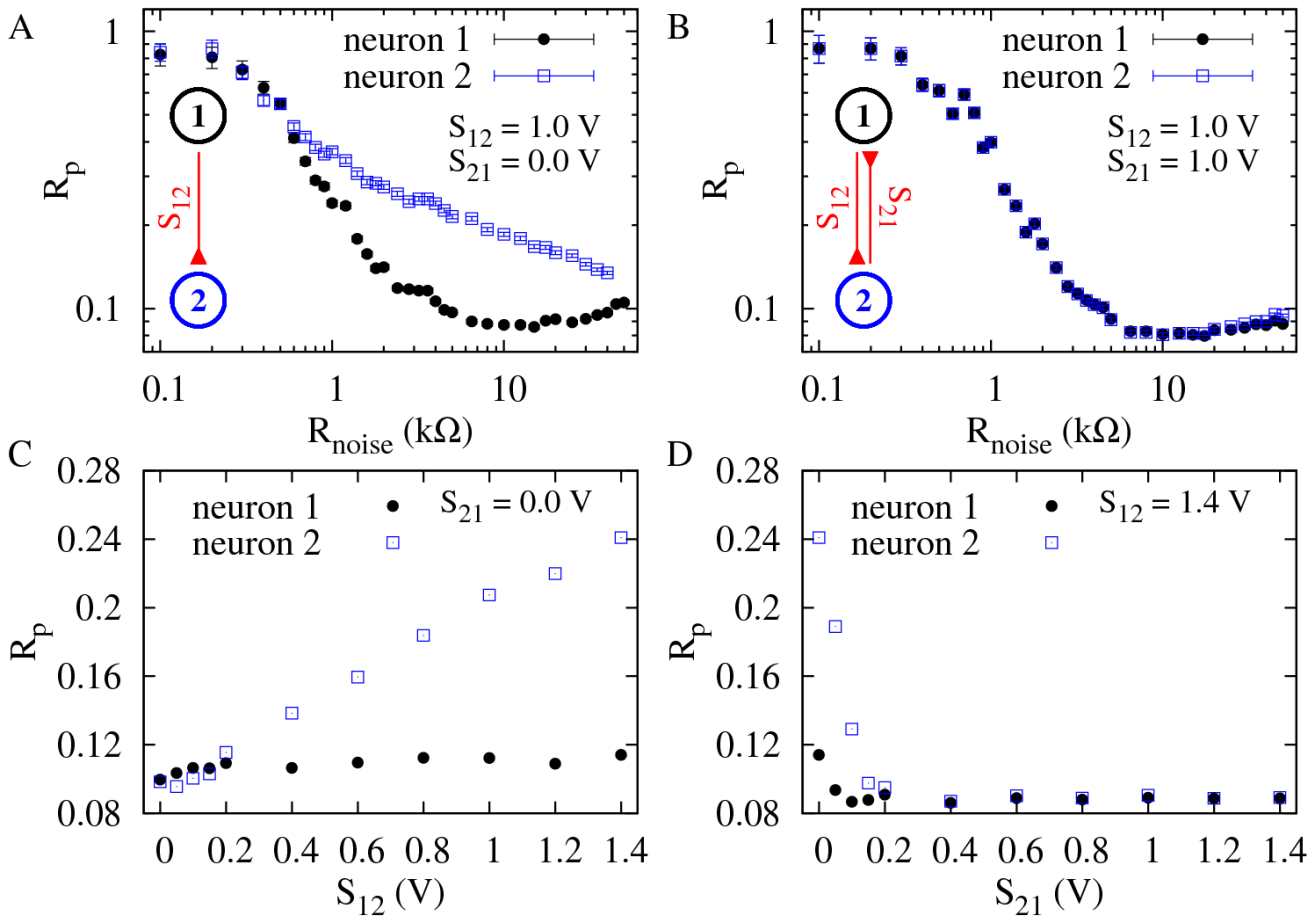
Incoherence parameter *R_p_* as a function of the noise intensity and synaptic amplitudes. (A) Neuron 1 (pre-synaptic) is connected to neuron 2 (post-synaptic) through a synaptic circuit with parameters 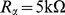, 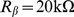 and synaptic amplitude 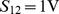. 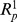 presents a minimum, as expected, but neuron 2 has increased incoherence 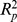. (B) Addition of an identical reciprocal connection leads to a coherence recovery in neuron 2. (C) 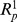 and 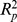 as functions of the synaptic amplitude 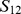 when 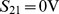 (unidirectional connection). (D) 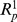 and 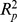 as functions of the synaptic amplitude 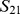 with 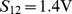. In (C) and (D), noise is set at the coherence resonance value for a single neuron (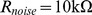).

To investigate the effects of symmetry in the synaptic coupling on the coherence of spike trains, we started by the asymmetrical extreme of connecting two neurons unidirectionally. As shown in Fig. 3A, the incoherence 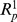 of the pre-synaptic neuron 1 exhibits a minimum as a function of the noise intensity, as is typical of CR [11], [16]. Due to the synapse from neuron 1 to neuron 2 (with synaptic amplitude 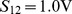), the spike trains of neuron 2 are less coherent than those of neuron 1 (Fig. 3A). This could be expected, since neuron 2 is receiving noise-induced spikes from neuron 1 in addition to its own (independent) noise source. In this simple scenario, the behavior of the pre-synaptic neuron is, as expected, unaffected by its outgoing synapse.

Keeping the synapse from neuron 1 to neuron 2, we added another synapse in the opposite direction, with the same synaptic amplitude (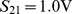). This reverted the effect of the single synapse, causing neuron 2 to reattain a minimum in the 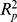 vs 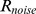 curve, as shown in Fig. 3B. This is perhaps counterintuitive, since one might expect that, by synaptically coupling the less coherent neuron-2 spike train with neuron 1, 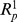 should increase. What happens, however, is that not only the coherence of neuron 1 is weakly affected, but also neuron 2 recovers its coherence. More importantly, it does so by means of an outgoing synapse.

We explored how the above phenomenon unfolds as we gradually change the synaptic strengths. We started with initially uncoupled electronic neurons (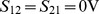) and noise intensity near its resonance value (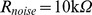). This choice of noise intensity was made in order to maximize the variation of the incoherence parameter 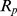 as the coupling is varied (see Fig. 3A and Fig. 3B). Increasing only the synaptic strength 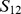, incoherence 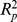 in the post-synaptic neuron increased monotonically, while 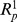 remained essentially unchanged (Fig. 3C). With 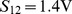 fixed, we then increased 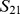, which led to a rapid increase in the coherence of neuron 2. Neuron 1, on its turn, showed a small decrease of incoherence (Fig. 3D), in a phenomenon similar to what has been reported in numerical simulations of symmetrically coupled neurons [14].

### Coherence depends weakly on synaptic symmetry for fast synapses

**Figure 4:**
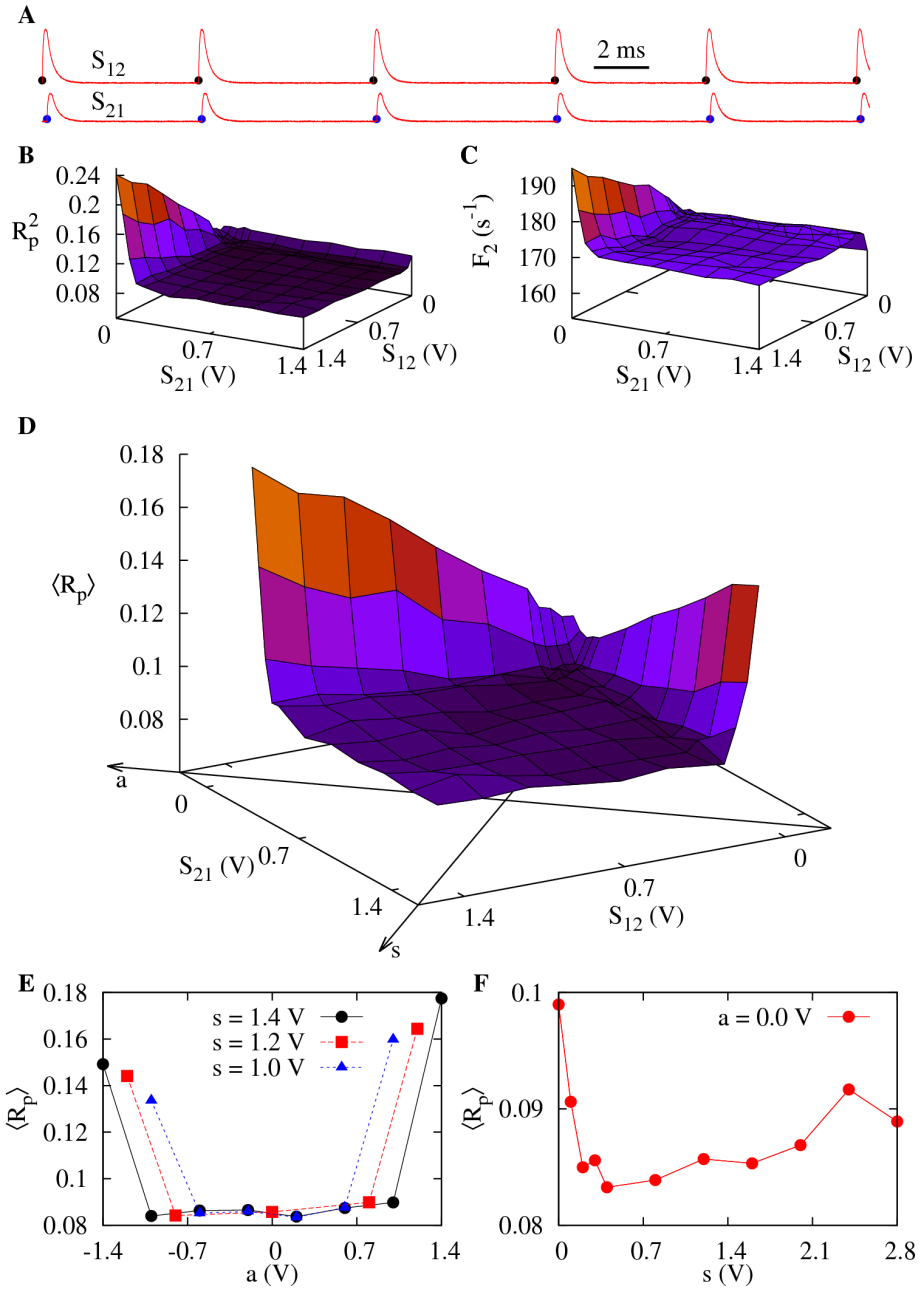
Influence of symmetry on CR for fast synapses. Noise intensities are set at the coherence resonance value (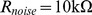) for each neuron. Synaptic parameters are 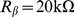 and 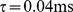. (A) Time series showing spike times for neuron 1 (black dots) and neuron 2 (blue dots) as well as the EPSP of the synapse connecting neuron 1 to neuron 2 with 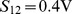 and the reverse synapse with 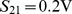. (B) Incoherence 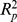 of neuron 2 as a function of both synaptic strengths. (C) Firing rate of neuron 2 as a function of the synaptic strengths. In (B) and (C) 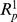 and 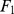 behave similarly if the indexes 1 and 2 are reversed. (D) Mean network incoherence 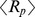 vs 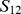 and 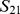. The symmetry and asymmetry axes are drawn on the horizontal plane. (E) 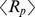 vs the asymmetry parameter *a*. (F) 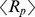 vs the symmetry parameter *s*.

The above results suggest that symmetry between the synaptic strengths 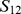 and 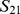 plays an important role in the spike train coherence of both neurons. To perform a thorough investigation of this phenomenon, we looked into the dependency of both 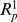 and 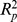 on 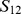 and 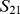 in a large region of the parameter space. In Fig. 4A we show the EPSP for both synapses with different synaptic strengths and the corresponding spike times on both neurons when the synaptic time scale is 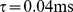 (in what follows, 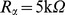 is kept fixed and 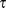 is controlled only by the resistance 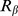). The dependency of the incoherence parameter 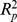 on the synaptic strengths is shown in Fig. 4B (of which Figs. 3C and 3D are cross-sections). The firing rate of neuron 2 was also measured as a function of the synaptic strengths and is shown on Fig. 4C. Note that in this case an increase in the excitatory synapse (with strength 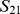) from neuron 2 induces a decrease in its own firing rate, which leads to an increase in the spike train coherence. The parameters 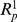 and 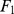 for neuron 1 behave in the same way if the indexes 1 and 2 are exchanged.

The total effect of the synaptic coupling on the system can be measured if we define the mean incoherence parameter 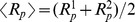. We show its dependence on 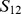 and 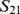 in Fig. 4D. This graph suggests that the incoherence parameter can be described as a function of symmetry and asymmetry parameters defined as 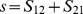 and 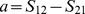 respectively. The *s* and *a* axes are also shown in Fig. 4D. Although for large values of 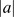 an increase in 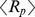 is observed, there is a large flat region in which no significant change in coherence is observed. The larger the symmetry *s*, the larger the flat region along the *a* axis (Fig. 4E). For fixed asymmetry *a*, increasing *s* causes an increase in the system coherence, as shown Figure 4F.

### Coherence depends strongly on synaptic symmetry for slow synapses

**Figure 5:**
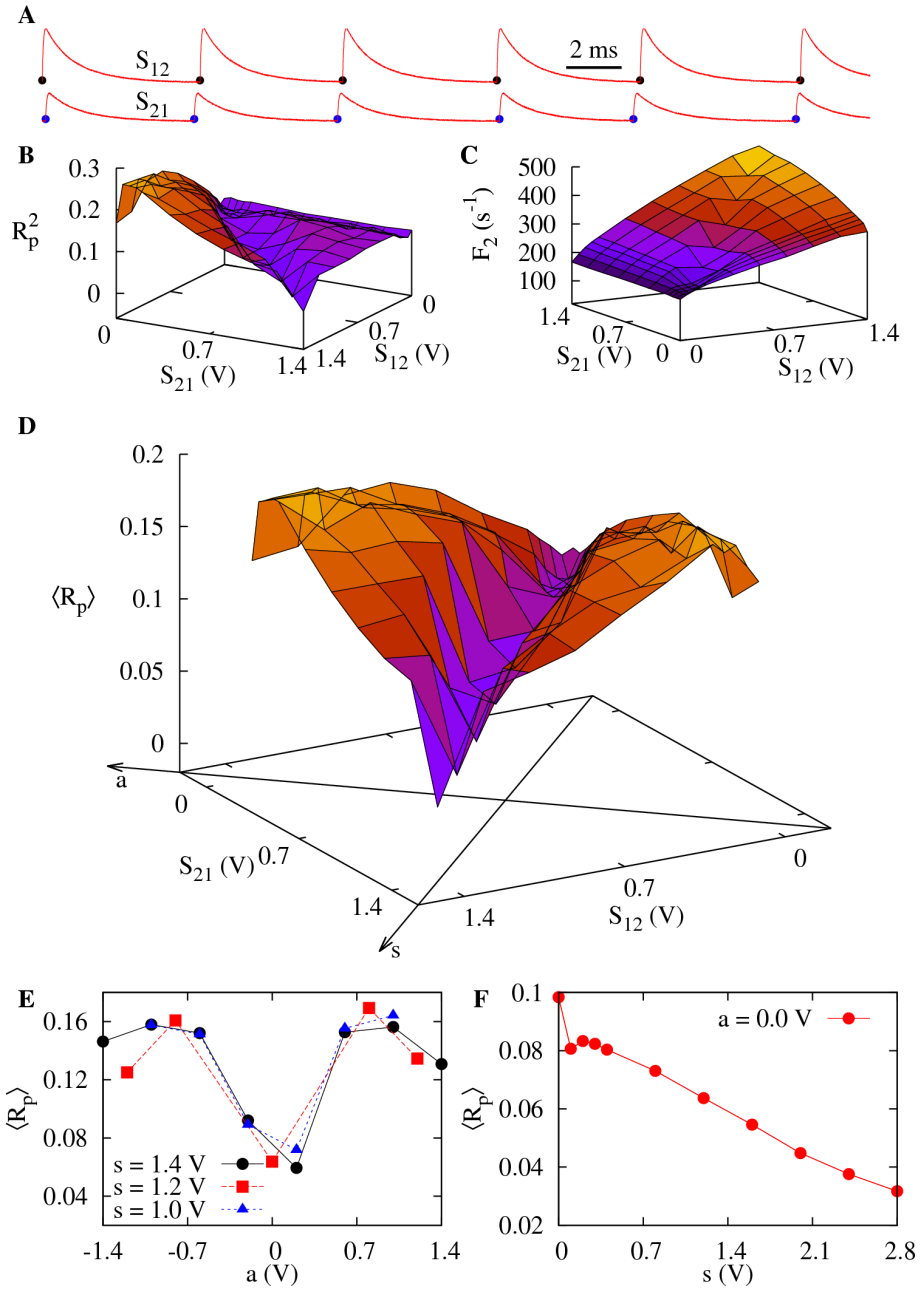
Influence of symmetry on CR for slow synapses. Same as Fig. 4, except that synaptic parameters are 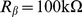 and 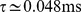).

The flat region in Fig. 4D might suggest that synaptic symmetry is not so relevant for coherence, after all. This scenario changes significantly, however, when the synaptic time scale increases. Figure 5 shows similar results to those of Fig. 4, but with 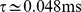 (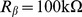). This 20% increase in the value of the synaptic time, as compared with the previous value, leads to a significant qualitative change in the EPSPs, as shown in Fig. 5A.

For these slower synapses, 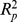 now displays a narrow valley of local minima as a function of 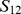 and 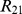 (Fig. 5B). If one revisits the situation in which 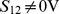 and 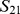 is increased from zero, once more an increase in coherence is observed in neuron 2 owing to an outgoing synapse (Fig. 5B). Differently from the scenario of the fast synapses (Figs. 4B and 4C), however, now the increase in the coherence of neuron 2 occurs with an *increase* in its firing rate, as shown in Fig. 5C.

The effect of synaptic symmetry on the overall coherence is much more pronounced for slower synapses, as shown in Fig. 5D. A much sharper minimum of 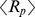 emerges near *a* = 0, regardless of the value of *s* (Fig. 5E). Furthermore, for fixed *a* = 0, an increase in symmetry *s* (which amounts to an increase in overall synaptic strength) leads to an increase in coherence, which attains values above those seen for uncoupled neurons (Fig. 5F).

### Comparison with computer simulations

We attempted to reproduce the above results in numerical simulations using the widely known FitzHugh-Nagumo model, as described in Eqs. 5. As in the experiments made with the electronic circuits, we set the model neurons in the excitable regime (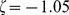 in Eq. 0) but close to its Hofp bifurcation (at 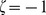), so that the system can fire under the influence of the Gaussian noise input 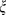 with intensity *D*. The two model neurons are connected through our model of the electronic synapse (Eq. 6) with a coupling coefficent *g* (see Eq. 5). The resulting EPSP 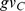 generated by a pre-synaptic spike is then added (alongside with the noise) to the variable 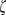 of the post-synaptic model neuron (see Eq. 5b). We find the noise intensity that yields the minimum of the incoherence parameter 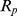 for the uncoupled case (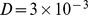) and then vary the synaptic strengths 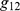 (from the model neuron 1 to model neuron 2) and 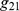 (from 2 to 1) measuring the mean incoherence parameter 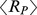 for each value of the pair 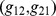 as we did with the electronic circuits. Note that the approach here is to measure the synaptic strength directly from the amplification factors 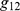 and 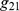 instead of measuring the amplitude of the EPSPs. The simulations were performed for two different values of the synaptic time scale 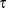, first for 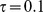 (fast synapse) and then for 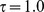 (slow synapse).

**Figure 6:**
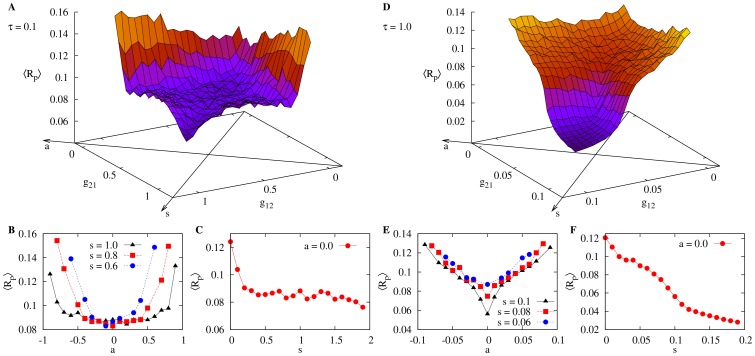
Influence of symmetry on CR for numerical simulations of synaptically coupled FitzHugh-Nagumo models. We have employed 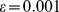, 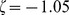 and 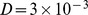 in all simulations. For 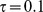 (fast synapses): (A) Mean network incoherence 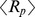 vs 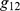 and 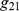. The symmetry and asymmetry axes are drawn on the horizontal plane. (B) 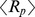 vs the asymmetry parameter *a*. (C) 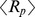 vs the symmetry parameter *s*. (D), (E) and (F) are the same as (A), (B) and (C), except that synapses are slower: 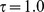.

The results of the numerical simulations are shown in Fig. 6. Comparing first Fig. 6A with Fig. 4D, we observe that the numerical model corroborates the results of the electronic circuits: with fast synapses, the dependence of the incoherence on the synaptic symmetry is weak. The mean incoherence parameter as a function of the symmetry parameter *s* and asymmetry parameter *a* is shown in Fig. 6B and Fig. 6C and there is good agreement with Fig.4E and Fig. 4F. Note that in the case of the computer simulations, we employ 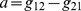 and 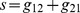.

Next we increase the synaptic time scale of the model (Fig. 6D) and compare the results with those of Fig. 5D. Although the shape of the surface obtained through the simulations does not exactly reproduce the one obtained experimentally with the electronic circuits, the dependence of the mean incoherence parameter on *a* and *s* keeps its main features for the case of slow synapses: the 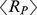 dependency on *a* is sharper than in the case of fast synapses (Fig. 6E). It also reaches smaller values when *s* increases, as compared to faster synapses (Fig. 6F).

## Conclusions

We have studied the effects of synaptic coupling between excitable electronic neurons on the coherence of their spike trains. We have shown that the coherence resonance observed in uncoupled neurons deteriorates in the post-synaptic neuron when the synaptic connection is unidirectional. Furthermore, coherence resonance is restored and strengthened when the synaptic loop is closed in a bidirectional coupling.

It is important to emphasize that, although this phenomenon may look similar to the synchronization between bidirectionally coupled spiking neurons [4], [18], here neurons are excitable and the only attractor of the system is a fixed point with both neurons quiescent. In this sense, it is interesting that the interplay between noise and synaptic coupling leads a post-synaptic neuron to regain its coherence by means of an outgoing synapse.

Furthermore, the mechanism by which this increase in coherence is attained depends on the synaptic time scale. With faster synapses, moving from a unidirectional coupling to bidirectional coupling by strengthening one of the synapses leads to an increase in coherence while firing rates decrease (despite the fact that all synapses are excitatory). For slower synapses, the same coherence increase is achieved with an increase in firing rates.

Overall, our results show that, for fast synapses, the average coherence of the spike trains can be maintained in a broad region of synaptic-strength parameter space. However, as synapses become slower, maximal coherence is achieved only in a much more restricted region, around the symmetry axis 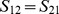. Along this axis, strengthening synaptic connections lead to an increase in coherence beyond the values attained by isolated neurons.

All the results above for electronic neurons were qualitatively reproduced with computer simulations of synaptically coupled FitzHugh-Nagumo models, suggesting the phenomenon is robust. It would be worth exploring whether it remains valid when neurons have a different excitability class, such as those near a saddle-node bifurcation [19]. Such type-I-excitable neurons can often be further reduced to simpler descriptions [20] which then might allow an analytical understanding of the results presented here.

Naturally, the ultimate test of our results would come from electrophysiological recordings of real neurons in which the symmetry of the synaptic coupling could be controlled. Although we are unaware of experiments in that direction, our results could also be useful to (or validated in) other systems not directly related to neuroscience, but where CR has been experimentally observed. The experimental setups range from semiconductor lasers [21] to the famous Belousov-Zabotinsky chemical reaction [22], recently also reaching nanoscopic scales in the ionic transport through single-walled carbon nanotubes [23]. If, for instance, applications on these setups depend on highly coherent states, then symmetry in the coupling may prove useful to overcome coherence degradation due to external factors.

The emergence of self-sustained activity, a recurrent theme in the field of excitable media [24], could also be the subject of future studies regarding the effects of coupling asymmetry. Even in our simple two-neuron network, for example, preliminary results suggest that increasing the time constant 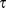 beyond the values used in this work can throw the system in an self-sustained attractor, despite the fact that both neurons are individually in a excitable state. Similar effects have been observed in a pair of electrically-coupled 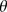-neurons [20] and are likely to be important in an electronic implementation of neuro-inspired artificial sensors, which are predicted to have maximal dynamic range and sensitivity at the transition to a self-sustained state [25].

Taken together, our results point to the importance of allowing for inhomogeneity in CR studies of coupled excitable elements. Our study of a coupled pair, where inhomogeneity is reduced to the asymmetry of the synaptic connections, can be regarded as a first step towards larger networks.
